# Solvent effect on the spectral properties of Neutral Red

**DOI:** 10.1186/1752-153X-2-19

**Published:** 2008-09-18

**Authors:** Muhammad A Rauf, Ahmed A Soliman, Muhammad Khattab

**Affiliations:** 1 Chemistry Department, P.O.Box 17551, UAE University, Al-Ain, UAE; 2 Chemistry Department, Cairo University, Cairo, Egypt

## Abstract

**Background:**

The study was aimed at investigating the effect of various solvents on the absorption spectra of Neutral Red, a dye belonging to the quinone-imine class of dyes. The solvents chosen for the study were water, ethanol, acetonitrile, acetone, propan-1-ol, chloroform, nitrobenzene, ethyleneglycol, acetic acid, DMSO and DMF.

**Results:**

The results have shown that the absorption maxima of dyes are dependent on solvent polarity. In non-hydrogen-bond donating solvents, solvation of dye molecules probably occurs via dipole-dipole interactions, whereas in hydrogen-bond donating solvents the phenomenon is more hydrogen bonding in nature. To estimate the contribution of the different variables on the wave number of the Neutral Red dye, regression analyses using the ECW model were compared with the π* scale model. This showed that the unified scale for estimating the solvent effect on the absorption of the Neutral Red dye is more adopted and more applicable than the π* scale model.

**Conclusion:**

Absorption maxima of dyes are dependent on solvent polarity. Solvation of dye molecules probably occurs via dipole-dipole interactions in non-hydrogen-bond donating solvents, whereas in hydrogen-bond donating solvents the phenomenon is more hydrogen bonding in nature. The unified scale for estimating the solvent effect on the absorption of Neutral Red dye is more adopted and more applicable than the π* scale model. This may be due to complications from both π-π* charge transfer interactions and incomplete complexation of the solute; these effects are averaged out in the derived β and π parameters and thus limit their applicability.

## Background

It is very well known that chemical processes are influenced by the properties of solvents in which they are carried out. These include the dipole moment, dielectric constant, and refractive index values. The most important property in this regard is the solvent polarity which can change the position of the absorption or emission band of molecules by solvating a solute molecule or any other molecular species introduced into the solvent matrix. A number of literature citations are available for the studies of simple organic molecules with regard to their interactions in different solvents [1-3]. Dye molecules on the other hand are complex organic molecules which might carry charge centers (as an integral part of their structure or because they are derived salts) and are thus prone to absorption changes in various media [4,5]. The structural complexity of dye molecules has drawn attention of many workers to understand their behavior in various media [6-8]. These changes are important to understand various physical-organic reactions of these macromolecules which have become important in different fields of pure and applied chemistry such as synthetic chemistry, extraction of dyes from solution, photodynamic therapy and chelation processes [9-11].

## Experimental

The dye used in this work was purchased from Sigma Chemicals and used as such. The solvents used in this work namely propan-1-ol, chloroform, acetonitrile, DMSO, ethanol, acetone, and DMF were purchased from Merck or Fluka and had a purity of > 99%. They were kept over molecular sieves 5Å prior to their use in this work. Stock solution of the dye was made in all these solvents and then diluting them appropriately with a given solvent. Absorption spectrum of each dye solution (1 × 10^-5 ^M) was recorded on a CARY 50 UV/VIS spectrophotometer, using a 1 cm quartz cell.

## Results and discussion

**Figure 1:**
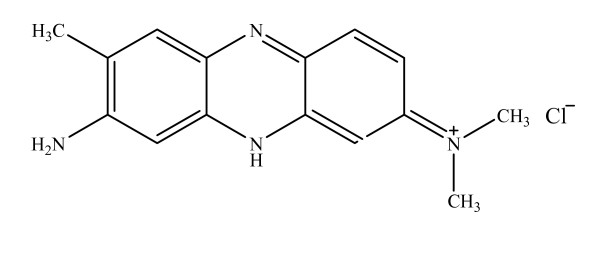
Chemical Structure of Neutral Red.

The present study deals with the solvent effect on the absorption spectra of Neutral Red (NR), which belongs to Quinone-Imine class of dyes. The molecular structure of this dye is shown in figure 1. Absorption spectrum of the dye solution was recorded in different solvents with the aim to probe the effects of various solvents and correlate various absorption parameters to dye spectra in various solvents. For this purpose solvents of different types were selected, firstly the non hydrogen-bond donating solvents (also called as non-HBD type of solvents) such as acetone, acetonitrile, nitrobenzene, DMF and DMSO; and secondly the hydrogen-bond donating solvents (also called as HBD type solvents) such as water, ethanol, acetic acid, ethyleneglycol, chloroform and propan-1-ol. The values of λ_max _of Neutral Red in these solvents are given in Table 1. One can see from this table that the absorption maximum of the dye is affected by solvent type and has a maximum shift of Δλ = 22 nm for the solvents used in this work. Thus this change in spectral position can be used as a probe for various types of interactions between the solute and the solvent.

**Table 1:** Absorption maxima of Neutral Red(NR) in various solvents and selective Kamlet-Taft solvent properties

Solvent	λ(nm)	π*	α	β	ε	n
Water	527	1.09	1.17	0.18	80.1	1.3330
Propan-1-ol	538	0.52	0.84	0.90	20.33	1.3856
Chloroform	522	0.58	0.44	0.0	4.81	1.4429
Acetonitrile	531	0.75	0.19	0.31	38.80	1.3440
DMSO	546	1.0	0.0	0.76	47.2	1.4790
Ethanol	537	0.54	0.83	0.77	24.55	1.3614
Acetone	525	0.71	0.08	0.48	20.70	1.3590
DMF	540	0.88	0.0	0.69	38.3	1.4305
Acetic acid	534	0.64	1.12	-	6.2	1.3710
Ethyleneglycol	539	0.92	0.9	0.52	38.7	1.4310
Nitrobenzene	544	1.01	0.0	0.39	32.0	1.5510

The analysis of solvent effect on spectral properties of dye solutions were carried out by using the spectral position in above mentioned solvents and correlating these with the Kamlet-Taft solvent properties namely, π*, α, β, n and ε, obtained from the literature [12,13]. Since the shift in λ_max _values with solvent type reflects dye-molecule interactions, an attempt was made to study this phenomenon in detail. Table 1 shows the essential solvent parameters required in this study along with the absorption maxima for each dye in these solvents. The spectral position of dye in various solvents has revealed interesting results. Since all the solvents used in this work were polar in nature, one would expect that the dye would bind more strongly to a more polar solvent and thus cause the spectra to shift to lower wavelengths. However, this is not seen from our results as λ_max _is lowest in the case of chloroform. This is due to the reason that all other solvents used in this work are more polar than chloroform and can engage more strongly in a solvent-solvent type of interaction. Thus their ability to interact with the dye molecules becomes less. On the other hand, chloroform which is less polar, can interact with the dye molecule in terms of dipole-dipole interactions, thereby resulting in a net stabilization of the ground state of the dye molecule, and hence one sees a hypsochromic shift in the spectrum in this solvent. On the other hand, the λ_max _value is shifted to lower energies in highly polar solvents such as DMSO because of strong solvent-solvent interaction or the specific interaction between the solvent and hydrogen from NH_2_group in the dye molecule.

**Figure 2:**
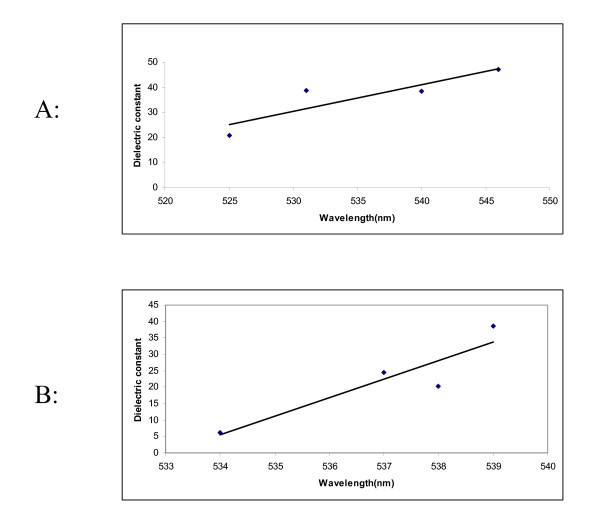
(A) Absorption shift of dye solution as a function of dielectric constant in non-hydrogen-bond donating solvents, (B) Absorption shift of dye solution as a function of dielectric constant in hydrogen bonding solvents.

**Figure 3:**
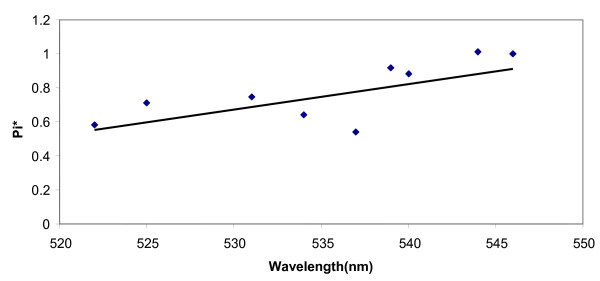
Absorption shift of dye solution as a function of solvent polarizability (π*).

A plot of λ_max _versus the dielectric constant values in various non-HBD and HBD solvents is shown in figure 2A and 2B. It can be seen from this figure that with increasing dielectric constant values, the spectrum is shifted to higher wavelength. The spectral changes observed in water were quite distinct from those in other solvents. The λ_max _of dye solution in water was found at lower wavelengths as compared to in other solvents although its dielectric constant is the highest among these solvents. This might be due to the formation of strong hydrogen bond between dye and water molecule. Thus different phenomena are present in various media. An increase in λ_max _values with π* (dipolarity/polarizability) as shown in figure 3 also indicates that dye interaction becomes different with increasing capability of a given solvent to form H bonds in solution.

The absorption data of dyes in various solvent was also analyzed in terms of various polarity scales. The first method involves the transformation of λ_max _(nm) of dyes in various solvents into molar transition energies {E_T_(dye), kcal/mole} by using the following relationship [14]

(1) E_T_(dye) = 28,591/λ_max_

The E_T_(dye) values signify transition energy which also reflects the stabilization of the dye in its ground state in a given solvent. This may be due to either hydrogen bond formation or dye-solvent interaction. Therefore, E_T_(dye) provides a direct empirical measure of dye solvation behavior. Again, from Table 2 one can see that E_T_(dye) is maximum in the case of chloroform as compared to other solvents. The rationale behind this is the same as described previously.

**Table 2:** Empirical parameters of solvent polarity

Solvent	E_T _(30) kcal/mol	E_T _(NR) kcal/mol	*f*(*ε*, *n*)	*g*(*n*)	*φ*(*ε*, *n*)
Water	62.8	54.25	0.91363	0.226851	1.367334
Propan-1-ol	50.7	53.14	0.77905	0.262207	1.303468
Chloroform	39.1	54.77	0.37245	0.300187	0.972823
Acetonitrile	55.4	53.84	0.86602	0.234279	1.334575
DMSO	45.1	52.36	0.84132	0.323779	1.488877
Ethanol	51.9	53.24	0.81293	0.245993	1.304918
Acetone	39.1	54.45	0.79028	0.244380	1.279042
DMF	43.2	52.95	0.83944	0.292021	1.423484
Acetic acid	51.7	53.54	0.49972	0.254245	1.004628
Ethyleneglycol	56.3	54.77	1.11158	0.292350	1.696281
Nitrobenzene	41.2	52.55	0.78171	0.369957	1.521691

**Figure 4:**
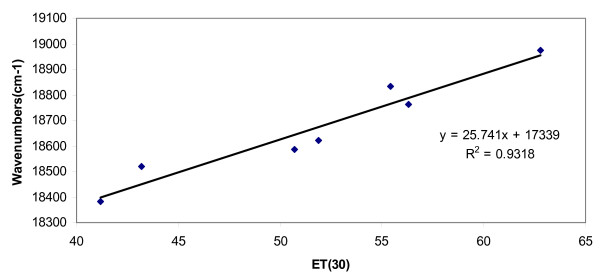
Plot of absorption value (in wavenumber) of Neutral Red in various solvents versus the E_T _(30) values.

The absorption values were also related to the solvent polarity parameter, namely E_T_(30), which also considers other interactions besides those of specific nature. The values of E_T_(30) were obtained from the literature for various solvents used in this work and are listed in Table 2[14]. Figure 4 shows the correlation between the absorption value (in wavenumber) and E_T_(30) for the dye studied in this work. A linear correlation of absorption energy covering a range of E_T_(30) indicates the presence of specific nature of interactions between the solute and solvents.

The spectral band shifts were also related to solvent parameter *φ*(*ε*, *n*) which is given as follows [15]

(2) *φ*(*ε*, *n*) = *f*(*ε*, *n*) + 2 *g*(*n*)

The function takes into account two important properties of the solvents namely the dielectric constant and the refractive index and is a sum of two independent terms namely *f*(*ε*, *n*) and *g*(*n*) which are given as follows

**Figure 5:**
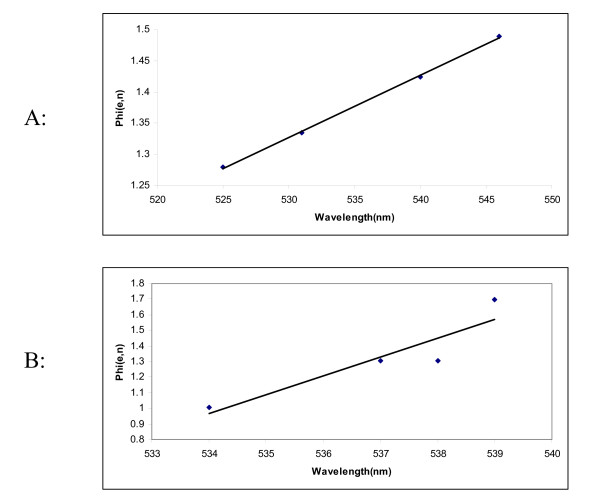
(A) Absorption shift of dye solution in non-hydrogen-bond donating solvents as a function of solvent polarity parameter *φ*(*ε*, *n*), (B) Absorption shift of dye solution in hydrogen bonding solvents as a function of solvent polarity parameter *φ*(*ε*, *n*).

(3) *f*(*ε*, *n*) = [(2*n*^2^+1)/(*n*^2^+2)] [{(*ε *- 1/*ε *+2)} - {(*n*^2^-1)/(*n*^2^+2)}]

(4) *g*(*n*) = 3/2 [(*n*^4^-1)/(*n*^2^+2)^2^]

where, *ε *is the dielectric constant and *n *is the refractive index and both these quantities reflect the freedom of motion of electrons in the solvent and the dipole moment of the molecules. Specific solvent effects occur by interactions of the solvent and the chromophores. Figure 5A and 5B shows the trend when the spectral position (λ_max_) of the dyes in various solvents (non-protic and protic) were plotted against the solvent polarity parameter *φ*(*ε*, *n*).

Since dye spectra are also influenced by the presence of a co-solvent, some studies on selective mixtures of solvents were also carried out and their results are hereby discussed. For these studies, two sets of solvents were selected. The first solvent mixture selected was ethanol and water mixture, which belongs to the HBD type of solvents, whereas, the second solvent mixture consists of acetone and ethanol, which belong to the HBD-non HBD type of solvents. The mixtures were prepared in various mole fractions containing a fixed amount of dye.

**Figure 6:**
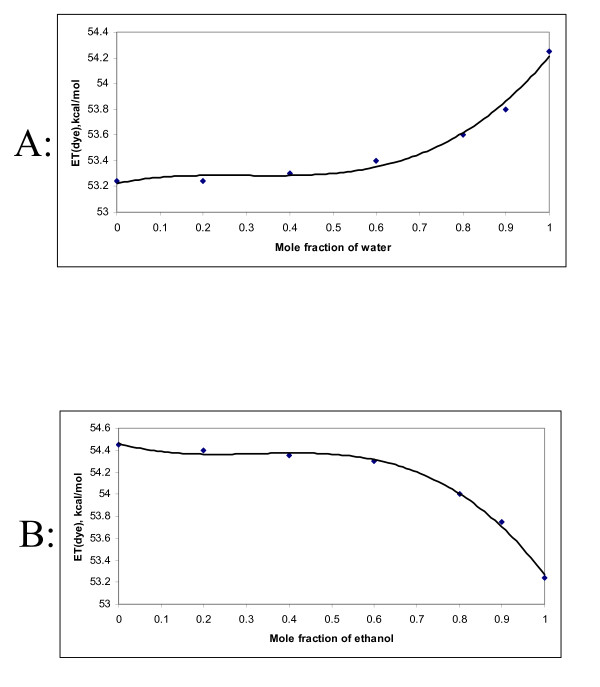
(A) E_T_(NR) as a function of mole fraction of water in water-ethanol mixture, (B) E_T_(NR) as a function of mole fraction of ethanol in acetone- ethanol mixture.

In ethanol-water mixtures, the spectra were found to shift towards red with increasing mole fraction of ethanol. These results show that the dye cation is preferentially solvated by the alcoholic component in all mole fractions in aqueous mixtures with ethanol. It is well known that water makes strong hydrogen-bonded nets in the water-rich region, which are not easily disrupted by the cosolvent [13]. This can explain the strong preferential salvation by the alcoholic component in this region since water preferentially interacts with itself rather than with the dye. In the alcohol-rich region, the alcohol molecules are freer to interact with the water and with the dye, since their nets formed by hydrogen bonds are weaker than in water. In this situation, the alcohol molecules can, to a greater or lesser extent, interact with water through hydrogen bonding. A change in the E_T_(NR) as a function of mole fraction of water in water-ethanol mixture is shown in figure 6A. The E_T_(NR) in the mixture was calculated by the method given in the literature [5].

On the other hand, in the case of acetone and ethanol mixtures, the spectra also shifted towards red with increasing amount of ethanol. The solvation of dye in non-HBD type of solvent mainly occurs through charge-dipole type of interaction, whereas in HBD type of solvent, the interaction also occurs by hydrogen bonding besides the usual ion-dipole interaction. In this situation, the methyl groups of acetone are responsible for the solvation of the dye. Thus in these solvent mixtures, increasing the amount of HBD solvent (ethanol in this case) shall break these interactions with the dye molecule, thereby shifting the spectra towards red. A similar behavior is reported in the literature for some other probe molecule [5]. Figure 6B shows the change in E_T_(NR) as a function of mole fraction of ethanol in acetone-ethanol mixture.

Great attention has been paid to the problem of solvent effect on spectral, chemical and reactivity data [16]. Kamlet considered the total solvent effect to be composed of three independent contributions; solvent polarity (π*), acidity(α) and basicity(β) for hydrogen bond acceptor(HBA) solvents. These contributions are gathered in one equation as follows:

(5) ν = ν_o _+ *s*π* + *a*α + *b*β

Where ν is the wave number at maximum absorption, *s, a *and *b *are regression factors, whose values depend on the extent of contribution of each solvent parameter (π*, α,β) to the predicted values ν'.

A unified scale of solvent polarities, taking into account both the non-specific and specific donor acceptor interactions of these solvents with solute probes, was introduced by Drago and co-workers and shown in its mathematical form below [17]:

(6) Δχ = E*_A _E_B _+ C*_A_C_B _+ S'P + W

where Δχ or χ is used as a value of physicochemical property measured in the specific solvent polarity; P is a measure of the susceptibility of the solute probe to solvation and W is the value of Δχ at S' = 0 value which provides a scale of non-specific solvating ability. Similarly, E_B _and C_B _are solvent parameters which are reported for the donors reacting with a wide range of acceptors in solvents of poor solvation property [18].

The unified scale of solvent polarities was expanded to include the very important class of polar hydrogen bonding solvents. Since these solvents are capable of undergoing both non-specific and specific donor-acceptor interactions with donor solute probes, the above equation modifies to:

(7) Δχ = E'_A _E*_B _+ C^'^_A_C*_B _+ S'P + W

The prime values represent parameters that are consistent with enthalpy based parameters of the *ECW *model [18-21].

To estimate the contribution of the different variables on the wave number of the Neutral Red dye; the coefficients in equations (5) and (7) were estimated using a multiple linear regression analyses (where Δχ in equation 7 is used as the wave number (ν) of the dye in the different solvents), and the results are shown in Tables 3, 4 and 5. The high R^2 ^value for the regression using *ECW *model compared with that of the π* scale model reflect the fact that the unified scale for estimating the solvent effect on the absorption of Neutral Red dye is more adopted and more applicable than the π* scale model. This may be due to complications from both π-π* charge transfer interactions and incomplete complexation of the solute; these effects are averaged out in the derived β and π parameters and thus limit their applicability [22]. DMF and DMSO were incompatible and this could be attributed to complications in the specific interactions which may arise due to a variety of bonding sites of different extent in these solvents.

**Table 3:** Neutral Red absorption (cm^-1^) in various solvents and some selective solvent properties

Solvent	ν(cm^-1^)	E'_A_	C'_A_	S'
Water	18975.33	1.91	1.78	3.53
Propan-1-ol	18587.36	1.28	0.83	2.66
Chloroform	19157.09	1.56	0.44	1.74
Acetonitrile	18832.39			
DMSO	18315.02			
Ethanol	18621.97	1.33	1.23	2.8
Acetone	19047.62			
DMF	18518.52			

**Table 4:** π*-scale parameters of Neutral Red

Effect	Coefficient	Std Error	Std Coef	Tolerance	t	P(2 Tail)	R^2^	R^2^_adjusted_
	19473.838 (ν_o_)	278.232	0	.	69.991	0	0.830	0.702
π*	-438.856 (s)	288.061	-0.322	0.952	-1.523	0.202		
α	63.216 (a)	135.746	0.098	0.969	0.466	0.666		
β	-805.858 (b)	191.874	-0.888	0.953	-4.2	0.014		

**Table 5:** ECW parameters of the Neutral Red as a new probe

Effect	Coefficient	Std Error	Std Coef	Tolerance	t	P(2 Tail)	R^2^
	18220.243 (W)	0	0	.	.	.	1.00
E'_A_	976.912 (E*_B_)	0	1.013	0.501	.	.	
C'_A_	90.525 (C*_B_)	0	0.187	0.042	.	.	
S'	-360.325 (P)	0	-0.956	0.05	.	.	

## Conclusion

Absorption maxima of dyes are dependent on solvent polarity. Solvation of dye molecules probably occurs via dipole-dipole interactions in non-hydrogen-bond donating solvents, whereas in hydrogen-bond donating solvents the phenomenon is more hydrogen bonding in nature. The unified scale for estimating the solvent effect on the absorption of Neutral Red dye is more adopted and more applicable than the ð π* scale model. This may be due to complications from both πð- π* charge transfer interactions and incomplete complexation of the solute; these effects are averaged out in the derived β and π parameters and thus limit their applicability.

## Competing Interests

The authors declare that they have no competing interests.
